# Effects of on- and off-year management practices on the soil organic C fractions and microbial community in a Moso bamboo (*Phyllostachys edulis*) forest in subtropical China

**DOI:** 10.3389/fpls.2022.1020344

**Published:** 2022-12-09

**Authors:** Zhiyuan Huang, Qiaoling Li, Xu Gai, Xiaoping Zhang, Zheke Zhong, Fangyuan Bian, Chuanbao Yang

**Affiliations:** ^1^ China National Bamboo Research Center, Key Laboratory of Bamboo Forest Ecology and Resource Utilization of National Forestry and Grassland Administration, Hangzhou, Zhejiang, China; ^2^ National Long-term Observation and Research Station for Forest Ecosystem in Hangzhou-Jiaxing-Huzhou Plain, Hangzhou, Zhejiang, China; ^3^ Key Laboratory of High Efficient Processing of Bamboo of Zhejiang Province, Hangzhou, Zhejiang, China; ^4^ Research Institute of Subtropical Forestry, Chinese Academy of Forestry, Hangzhou, Zhejiang, China; ^5^ Engineering Research Center of Biochar of Zhejiang Province, Hangzhou, Zhejiang, China

**Keywords:** Bamboo, on- and off-year, C sequestration, bacteria, fungi

## Abstract

On- and off-year management practices are usually adopted in Moso bamboo (*Phyllostachys edulis*) forests to achieve higher productivity. However, little is known about the effects of these management practices on soil C sequestration and microbial community structure. In the present study, soil nutrient content, organic C fractions, and bacterial and fungal communities were comparatively investigated in on- and off-year bamboo stands. The results showed that soil organic C (SOC), alkali-hydrolyzable N (AN), and available P (AP) in the on-year were significantly lower (*p* ≤ 0.05) than those in the off-year. Among the different soil organic C fractions, easily oxidizable organic C (EOC), microbial biomass C (MBC), Ca-bound SOC (Ca-SOC), and Fe/Al-bound SOC (Fe/Al-SOC) also had significantly higher contents in the off-year than in the on-year, with MBC and EOC decreasing by 56.3% and 24.5%, respectively, indicating that both active and passive soil organic C pools increased in the off-year. However, the alpha diversities of both soil bacteria and fungi were significantly lower in the off-year soils than in the on-year soils. The bacterial taxa Actinobacteria, Planctomycetes, WPS-2, *Acidothermus, Candidatus_Solibacter, Burkholderia-Caballeronia-Paraburkholderia*, and *Candidatus_Xiphinematobacter* were increased in off-year soils relative to on-year soils. Meanwhile, fungal taxa Ascomycota, *Mortierella*, *Hypocrea*, *Cryptococcus*, *Clitopilus*, and *Ceratocystis* were significantly increased in on-year soils. Soil pH, SOC, AP, MBC, EOC, and Ca-SOC were significantly correlated with bacterial and fungal communities, with soil pH being the most important driving factor for the shift in bacterial and fungal communities. Our findings showed that the studied bamboo forest possessed an inherent restorative ability in the off-year, which can reverse the soil nutrient and C depletion in the on-years and ensure soil fertility in the long term.

## 1 Introduction

Bamboo is a key forest resource and is mainly distributed in tropical, subtropical, and temperate areas (Scurlock et al., 2000). The total area of bamboo forests worldwide is approximately 31.5 million ha and accounts for approximately 0.8% of the global forested area (FAO, 2010). In China, bamboo forests cover more than 6 million ha, 73.8% of which are Moso bamboo forests (Wang et al., 2013; Song et al., 2016a). Moso bamboo is a large uniaxial bamboo; it completes its body size growth in 40 days, during which it increases its C storage 45 times. The annual aboveground C sequestration rate in a Moso bamboo forest was found to be 2.39 times greater (8.13 ± 2.15 Mg ha^-1^ yr^-1^) than that in a Chinese fir forest (3.35 ± 2.02 Mg ha^-1^ yr^-1^) due to its unique growth features (Yen and Lee, 2011). Therefore, it has a high C sequestering capacity.

Moso bamboo forests have on-year and off-year periods, depending on the production of new bamboo shoots. The On-year period refers to the calendar year with high bamboo shoot production, whereas the off-year period refers to the year with lower bamboo shoot production (Li et al., 1998; Zhou et al., 2011; Chen et al., 2018). During its growth, bamboo shoots grow from March to May in the on-year and the leaves do not wither, while in April-May of the off-year, Moso bamboo shoots rarely grow, and all the leaves gradually turn yellow and wither, after which new leaves start growing (Gratani et al., 2008; Song et al., 2016a). Bamboo leaves are biennial, except in newly planted bamboo (Li et al., 1998). In many cases, on- and off-years alternate, forming a regular biennial cycle (Li et al., 1998). Moso bamboo forests are usually managed according to the growth pattern of on- and off-years. This management is called on- and off-year management and includes digging the bamboo shoots and felling bamboo plants in the on-year and nourishing the bamboo plants in the off-year (Chen et al., 2018). Studies have shown that Moso bamboo forests consume a lot of different nutrient elements in the soil during different growth periods (Li et al., 2000; Wu et al., 2006; Song et al., 2016a; Zhang et al., 2021a). Other studies have shown that N, P, and K concentrations in Moso bamboo roots, stems, and leaves change significantly during the bamboo shoot growing season and leaf renewal (Ito et al., 2014; Umemura and Takenaka, 2014). This indicates that in Moso bamboo forests, different elements in the soil play different roles in different years. On- and off-year Moso bamboo forests have diverse physiological and ecological properties, which consequently have different effects on soil nutrient uptake and utilization as well as on C fraction cycling (Song et al., 2016b; Zhou et al., 2019). Studies had been shown that soil organic C (SOC) content has an important relationship with processes such as nutrient cycling and soil microbial metabolism in forests (Ren et al., 2018; Yang et al., 2021; Zhang et al., 2021b). The amount of the SOC pool and the pace of mineralization, coupled with the activities of C-cycling enzymes, are significantly impacted by the change in forest management types (Lin et al., 2018). For example, Li et al. (2012b) found that fertilization affected SOC sequestration in paddy soils between no-till and conventional tillage practices. Li et al. (2013) showed that SOC stocks and water soluble organic C, hot-water soluble organic C, microbial biomass C (MBC) and readily oxidizable C concentrations decreased with time under intensive management relative to conventional management practices in Moso bamboo forests. However, studies on the effects of on- and off-year management practices on the C sequestration in Moso bamboo forests have rarely been conducted.

The physical, chemical, and biological components of soil depend on soil organic C (Bhogal et al., 2009; Esmaeilzadeh and Ahangar, 2014). C intake *via* litter breakdown, root turnover, animal feces, and other sources, along with C outflow through soil respiration, determines the level of SOC at any given time (Cui et al., 2005). According to several studies, particular SOC fractions are sensitive indicators of evaluating the advantages and disadvantages of various management strategies and are crucial for maintaining soil quality, such as soil active organic C (Chan et al., 2001; Yang et al., 2005). Soil active organic C is a highly active component of SOC with a high turnover rate and is easily utilized by soil microbes. Based on the concept of soil structure hierarchy, different levels of soil structure organization are supported by different forms of SOC. For this reason, some studies have suggested that compared to the general SOC, soil active organic C, as assessed by various approaches, is more susceptible to environmental changes (Díaz-Raviña et al., 1993; Holt, 1997). Soil MBC is the most active component of soil organic matter, and its proportion in the soil carbon pool is small, generally accounting for only 1%-4% of the SOC (Sparling, 1992), but it is a large source and stock for effective soil nutrients (Fan and Hao, 2003). MBC is an easily available nutrient pool and a driving force for organic matter decomposition and C and N mineralization in soils, and is closely related to the nutrient cycle of C, N, P and S in soils (McGill et al., 1986). Compared with SOC, MBC responds quickly to changes in soil management practices such as tillage and straw culture and can be an early indicator of changes in SOC and an indicator of changes in active organic C (Wang et al., 2004; Ferreira et al., 2016).Therefore, studying active organic C pools would help elucidate the mechanisms involved in the turnover of SOC pool under different management practices.

Soil microorganisms respond differentially to soil C dynamics as a consequence of the differences in plant diversity and organic matter content among different land-use change types (Li et al., 2012a; Deng et al., 2016). However, the direction and magnitude of these responses are poorly understood. For example, Fontaine et al. (2007) showed that high amounts of plant residue inputs may lower the efficiency with which microorganisms consume C or degrade soil organic matter, consequently reducing the soil C storage. In contrast, some studies have demonstrated that increasing plant variability or plant residue inputs can enhance the soil C storage from new C by modifying soil microbial proliferation (Lange et al., 2015; Tardy et al., 2015). Nonetheless, contradictory findings have indicated that soil microorganisms are involved in crucial ecological processes in a changing environment, such as C and N cycling. Furthermore, several microbial species, including Ascomycota, Basidiomycota, and Proteobacteria species, contribute to the breakdown of soil organic matter, resulting in changes in SOC fractions and, eventually, soil CO_2_ outflow (Goldfarb et al., 2011; Tardy et al., 2015). However, it is unclear how changes in SOC fractions can be effectively explained by changes in microbial assemblages (Deng et al., 2016; Xiao et al., 2017).

Therefore, understanding the nutrient and C dynamics during on- and off-year management practices is not only important for shedding light on the sustainable management of bamboo forest ecosystems, but also for improving the prediction of C balances when assessing the effects of on- and off-year management practices on the SOC pool. The present study aimed to evaluate the effects of on- and off-year management practices on the SOC quality and microbial functionality in a Moso bamboo forest. Our specific goals were to: (1) comparatively investigate the effects of on- and off-year management practices on soil C fractions and pertinent soil properties; (2) describe the shift in soil bacterial and fungal community structure under on- and off-year management; and (3) investigate the relationships between the change in the bacterial and fungal community and the shift in C fractions and assess the land-use sustainability of the on- and off-year management model.

## 2 Materials and methods

### 2.1 Site description

The study site was located in Tianhuangping Town, Anji County, Zhejiang Province, China (30°29′ N, 119°42′ E). The area has a mid-latitude subtropical monsoon climate, with an average annual temperature of 17°C and an average annual rainfall of approximately 1300 mm. The average annual sunlight duration is 1946 h, with 230 frost-free days (Li et al., 2014). The study area has a low, mountainous and hilly landscape. The soil of the sample site was classified as ferric luvisol (FAO, 1990), which is slightly acidic, and the soil matrix was determined to be a mixture of silt and fine sand (Yang et al., 2019).

The on- and off-year Moso bamboo forest was originally a natural evergreen broad-leaved forest, which was transformed into a pure Moso bamboo forest through human modification and nurturing after the mid-1960s. Subsequently, on- and off-year management practices started to be implemented. The shrub layer under the Moso bamboo forest mainly consisted of three species: bilberry, hickory pepper, and raspberry. The herb layer consisted of five species, namely dog’s spine fern, hare’s umbrella, lox, white flower septoria, and Baoduo grass.

### 2.2 Experimental design and soil sampling

A 20 m × 10 m sample plot with the same slope, slope surface, and elevation was set up in each on- and off-year Moso bamboo forest sample plot. Using the S-shaped sampling method, 10 sample points were set up at opposite locations of the two sample plots, and five Moso bamboo were selected near each sample point. Their rhizosphere soil was collected and mixed into one sample, and this process was repeated 10 times to collect a total of 20 soil samples. Fresh soil samples were sieved through a 2 mm sieve, partially air-dried for the determination of soil physicochemical properties and organic C fractions, and partially stockpiled at -80°C for soil microbial community characterization.

### 2.3 Basic soil properties

Soil pH was determined with a pH meter with a soil to water ratio of 1:2.5 (w/v). Soil alkali-hydrolyzable N (AN) was determined by the alkali-diffusion method (Bremner et al., 1996). Soil available P (AP) was determined with 0.03 mol·L^-1^ NH_4_F and 0.025 mol·L^-1^ HCl using the method described by Bray and Kurtz (1945). Soil available K (AK) was extracted using 1 mol·L^-1^ ammonium acetate solution and determined using a flame photometer (XP BWB, UK). Soil organic C (SOC) was determined using a TOC analyzer (Multi N/C 3100, Analytik Jena, Germany).

### 2.4 Extraction and analysis of the SOC fraction

Referring to the categorization technique of organic carbon fractions employed by Gai et al. (2021), five C fractions (namely easily oxidized organic C (EOC), dissolved organic C (DOC), MBC, Ca-bound soil organic C (Ca-SOC), and Fe/Al-bound soil organic C (Fe/Al-SOC)) were recovered from the SOC pool using chemical procedures. Oxidation with 333 mmol·L^-1^ KmnO_4_ was used to measure EOC (Blair et al., 1995). In brief, the air-dried soil containing 30 mg of C was weighed into a 50-mL centrifuge tube, and 25 mL of 333 mmol·L^-1^ KmnO_4_ solution was added, shaken at 200 rpm for 1 h, and then centrifuged at 4000 rpm for 5 min. The absorbance of the diluted solution was measured spectrophotometrically at 565 nm after diluting the supernatant by a ratio of 1:250 with deionized water. Finally, the EOC content was calculated based on the absorbance. DOC was extracted using 0.5 mol·L^-1^ K_2_SO_4_ and determined by a TOC analyzer (Bolan et al., 1996). The chloroform fumigation and extraction method was used to determine MBC (Vance et al., 1987). The technique outlined by Xu and Yuan (1993a) was used to determine the contents of Ca-SOC and Fe/Al-SOC in the samples. In brief, 2 g of air-dried soil was weighed and placed in a 100-mL centrifuge tube, and 20 mL of 0.5 mol·L^-1^ Na_2_SO_4_ solution was added, shaken at 180 rpm for 2 h, left for 24 h, and then centrifuged for 10 min at 3000 rpm. After repeating the above procedure several times, the supernatant was collected. A TOC analyzer was used to detect the amount of Ca-SOC in the supernatant. Finally, 20 mL of 0.1 mol-L^-1^ NaOH and 0.1 mol·L^-1^ Na_4_P_2_O_7_·10H_2_O were combined with the residue, centrifuged, and the content of Fe/Al-SOC in the supernatant was determined again by a TOC analyzer.

### 2.5 Bacterial and fungal communities analysis

According to the manufacturer’s protocol, DNA was extracted from the soil samples using the E.Z.N.A.^®^ Soil DNA Kit (D5625, Omega, Inc., USA). The 341F-805R (5′-CCTACGGGNGGCWGCAG-3′/5′-GACTACHVGGGTATCTAATCC-3′) and ITS1FI2-ITS2 (5′-GAACCWGCGGARGGATCA-3′/ 5′-GCTGCGTTCTTCATCGATGC-3′) primer sets were used to amplify the bacterial 16S V3-V4 region and fungal ITS2 genes. Amplicon synthesis, library construction, and Illumina NovaSeq sequencing (2 × 250 bp) were performed by LC-Bio Technology Co., Ltd. (Hangzhou, China). FLASH (Magoc and Salzberg, 2011) was used to construct paired-end 16s and ITS1 sequences, which were subsequently quality-trimmed and length-filtered using Fqtrim. DADA2 (Callahan et al., 2016) was used to construct the amplicon sequence variant (ASV) table, which was then allocated to the proper taxon using the QIIME 2 plugin (Bolyen et al., 2019). Taxonomy was assigned against the SILVA (release 132, https://www.arb-silva.de/documentation/release-132/) (Quast et al., 2012) and Unite (V8 released on 02.02.2019) databases (Abarenkov et al., 2010). The samples were rarefied to 51,038 sequences for bacterial communities and 38,330 sequences for fungal communities.

### 2.6 Statistical analysis

IBM SPSS (version 22.0; Chicago IL, USA) was used for the statistical analysis of soil chemical properties and organic C fractions. Significant differences among the soil samples were tested using an independent samples t-test, with *p* ≤ 0.05 determined as significant. Data are presented as mean ± SD. The ‘microeco’ package in R was used to compute alpha indices and perform the principal coordinate analysis (PcoA) (Liu et al., 2021), and the ‘vegan’ (Oksanen et al., 2019) package in R was used to perform the redundancy analysis (RDA) and assessed the effects of soil factors on the bacterial and fungal communities. The significant soil parameters were determined using the ‘envfit’ function in the vegan package. Spearman correlation analysis and Mantel test were performed using the ‘ggcor’ package in R (Huang et al., 2020). Random forest model (Breiman, 2001) was used to identify the key predictors of soil microbial communities using the ‘randomForest’ (Liaw and Wiener, 2002) package in R, and the significance of the model and each predictor were determined using the ‘rfUtilities’ (Evans and Murphy, 2019) and ‘rfPermute’ (Archer, 2016) packages, respectively. Linear regressions were visualized using the R ‘basicTrendline’ package (Mei et al., 2018).

## 3 Results

### 3.1 Soil properties

On- and off-year changes significantly affected the pH, SOC, AN, AP, and AK contents of rhizosphere soil in the Moso bamboo forest (*p* ≤ 0.05; **Table 1**). Soil pH and AK were significantly higher (*p* ≤ 0.05) in the on-year Moso bamboo stands than in the off-year Moso bamboo stands, whereas the opposite trend was observed for SOC, AN, and AP (*p* ≤ 0.05).

**Table 1:** Soil basic properties at the on- and off-year Moso bamboo forests.

Treatment	pH (H_2_O)	SOC (g·kg^-1^)	MBC/SOC (%)	AN (mg·kg^-1^)	AP (mg·kg^-1^)	AK (mg·kg^-1^)
Off-year	4.72 ± 0.05b	29.13 ± 2.90a	1.66 ± 0.67a	218.19 ± 30.67a	5.76 ± 1.03a	315.47 ± 54.79b
On-year	4.95 ± 0.05a	22.94 ± 2.35b	0.92 ± 0.61b	192.75 ± 22.31b	4.53 ± 0.36b	377.47 ± 40.31a

Different lowercase letters within columns indicate significant differences at p ≤ 0.05. Data are presented as mean ± SD, n = 10. SOC, soil organic C; MBC/SOC, the ratio of microbial biomass C to soil organic C, namely, microbial quotient; AN, alkali-hydrolyzable N; AP, available P; AK, available K.

### 3.2 Soil organic C fractions

**Figure 1:**
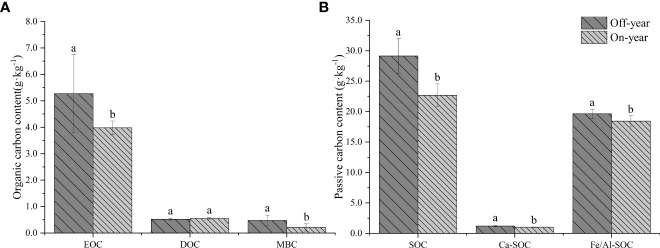
Soil active organic carbon **(A)** and passive organic carbon **(B)** content in the on- and off-year Moso bamboo forest. Different lowercase letters indicate significant differences among different distances by independent samples t-test (*p* ≤ 0.05). Error bars indicate standard deviation (n = 10). EOC, easily oxidized organic C; DOC, dissolved organic C; MBC, microbial biomass C; SOC, soil organic C; Ca-SOC, Ca-bound soil organic C; Fe/Al-SOC, Fe/Al-bound soil organic C.

SOC fractions were significantly different between on- and off-year stands (*p* ≤ 0.05) (**Table 1**; **Figure 1**). Soil EOC and MBC contents were significantly higher in the off-year Moso bamboo stands than in the on-year Moso bamboo stands (*p* ≤ 0.05). The MBC/SOC was 1.65% for off-year moso bamboo stands and 0.93% for on-year moso bamboo stands, with significant differences between them (*p* ≤ 0.05). However, difference in the DOC content between the two years was not significant (*p* > 0.05; **Figure 1A**). In contrast, DOC was generally much lower than EOC, accounting for 9.9% to 13.6% of the corresponding EOC.

The on- and off-year management practices significantly affected the passive C pool composition (*p* ≤ 0.05) (**Figure 1B**). Thus, Fe/Al-SOC was much higher than the corresponding Ca-SOC (specifically, 16.37 to 18.08 times higher). In general, the Ca-SOC and Fe/Al-SOC contents were significantly higher in the off-year Moso bamboo stands than in the on-year Moso bamboo stands (*p* ≤ 0.05).

### 3.3 Bacterial and fungal community alpha diversities

**Figure 2:**
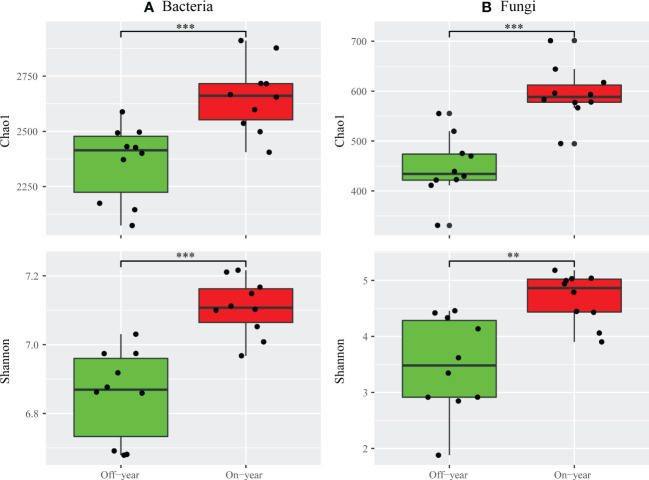
Alpha diversity indices of bacterial **(A)** and fungal **(B)** taxa for the on- and off-year soil samples from Moso bamboo plantations. ***p ≤ 0.001; **p ≤ 0.01; ns, *p* > 0.05.

The Chao1 and Shannon indices were used to evaluate the alpha diversities of bacterial and fungal communities (**Figure 2**). Compared with the off-year, the on-year significantly increased the Chao1 and Shannon indices of bacterial and fungal communities (*p* ≤ 0.05).

### 3.4 Compositions of bacterial and fungal communities

**Figure 3:**
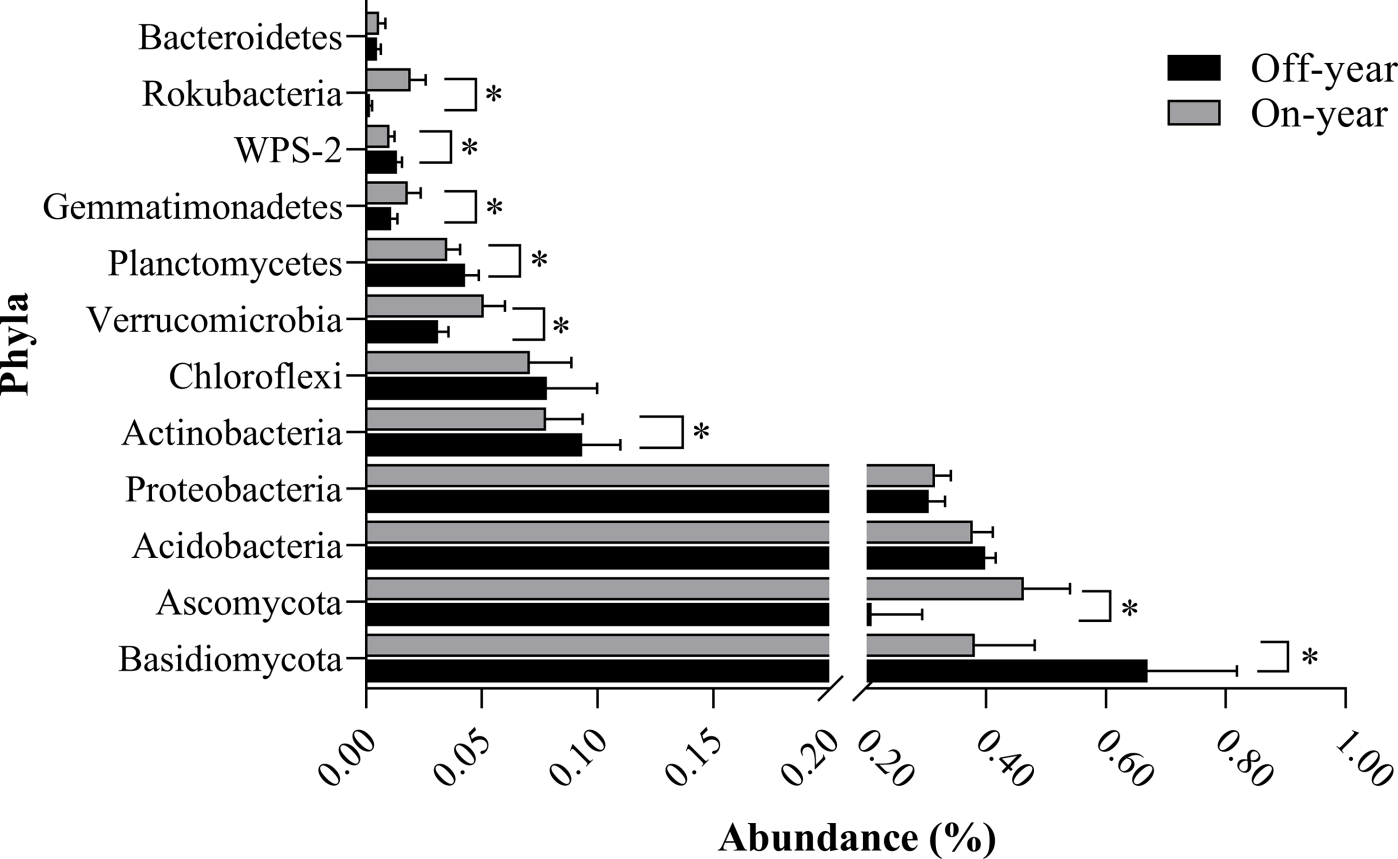
Bacterial and fungal compositions at the phylum level in the on- and off-year Moso bamboo forest soil. Bacterial and fungal phyla with an average relative abundance of greater than 0.5%. *p ≤ 0.05.

The most abundant bacterial phylum in the investigated soil samples was Acidobacteria, accounting for an average of 38.83% of the total sequences, followed by Proteobacteria, Actinobacteria, Chloroflexi, Verrucomicrobia, Planctomycetes, Gemmatimonadetes, WPS-2, Rokubacteria, and Bacteroidetes, accounting for 30.95%, 8.56%, 7.45%, 4.10%, 3.90%, 1.45%, 1.17%, 1.05%, and 0.52% of the total sequences, respectively (**Figure 3**). Within the fungal communities, Basidiomycota and Ascomycota were the dominant taxa, accounting for 52.57% and 33.60% of the total sequences, respectively. At the genus level, 13 bacterial and 12 fungal genera with an average relative abundance of > 0.5% were detected (**Table 2**).

**Table 2:** Relative abundances of the dominant bacterial and fungal genera in the on- and off-year Moso bamboo forest soil.

	Phylum	Genus	Off-year	On-year	*p-*Value
Bacteria	Actinobacteria	*Acidothermus*	4.73% ± 1.36%	3.33% ± 0.76%	0.011
	Acidobacteria	*Candidatus_Solibacter*	3.77% ± 0.39%	3.26% ± 0.25%	0.003
	Proteobacteria	*Acidibacter*	3.65% ± 0.65%	3.27% ± 0.31%	0.114
	Acidobacteria	*Bryobacter*	1.96% ± 0.29%	1.98% ± 0.41%	0.906
	Verrucomicrobia	*Candidatus_Udaeobacter*	0.72% ± 0.30%	2.88% ± 0.81%	0.000
	Proteobacteria	*Burkholderia-Caballeronia-Paraburkholderia*	1.74% ± 0.61%	1.23% ± 0.30%	0.031
	Proteobacteria	*Rhodoplanes*	1.17% ± 0.18%	1.18% ± 0.17%	0.931
	Proteobacteria	*Bradyrhizobium*	0.85% ± 0.15%	1.28% ± 0.39%	0.006
	Verrucomicrobia	*Candidatus_Xiphinematobacter*	1.02% ± 0.33%	0.73% ± 0.20%	0.030
	Proteobacteria	*Pajaroellobacter*	0.87% ± 0.26%	0.82% ± 0.22%	0.627
	Acidobacteria	*Candidatus_Koribacter*	0.67% ± 0.20%	0.84% ± 0.34%	0.187
	Verrucomicrobia	*ADurb.Bin063-1*	0.46% ± 0.16%	0.81% ± 0.23%	0.001
Fungi	Basidiomycota	*Camarophyllopsis*	8.02% ± 16.87%	0.91% ± 2.12%	0.217
	Zygomycota	*Mortierella*	2.70% ± 1.53%	5.41% ± 1.77%	0.002
	Ascomycota	*Cladophialophora*	2.26% ± 1.68%	2.63% ± 0.58%	0.524
	Basidiomycota	*Clavulinopsis*	2.71% ± 2.54%	2.17% ± 5.65%	0.785
	Ascomycota	*Hypocrea*	1.38% ± 1.27%	2.67% ± 1.11%	0.026
	Basidiomycota	*Entoloma*	1.82% ± 2.18%	1.84% ± 2.20%	0.987
	Basidiomycota	*Cryptococcus*	0.93% ± 0.62%	1.92% ± 0.83%	0.007
	Basidiomycota	*Clitopilus*	0.45% ± 0.27%	2.15% ± 1.52%	0.006
	Ascomycota	*Ceratocystis*	0.03% ± 0.03%	1.67% ± 1.75%	0.016
	Basidiomycota	*Agaricus*	1.69% ± 3.89%	0.00% ± 0.01%	0.204
	Basidiomycota	*Conocybe*	0.02% ± 0.03%	1.31% ± 4.01%	0.335
	Basidiomycota	*Trechispora*	0.92% ± 1.26%	0.26% ± 0.34%	0.141

At the phylum level, the abundances of the bacterial phyla Rokubacteria, Gemmatimonadetes, and Verrucomicrobia were significantly increased (*p* ≤ 0.05) while those of WPS-2, Planctomycetes, and Actinobacteria were significantly decreased (*p* ≤ 0.05) in the on-year samples compared with those in the off-year samples (**Figure 3**). The bacterial genera *Candidatus_Udaeobacter, Bradyrhizobium, and Adurb.Bin063-1* were significantly increased (*p* ≤ 0.05), whereas *Acidothermus, Candidatus_Solibacter, Burkholderia-Caballeronia-Paraburkholderia*, and *Candidatus_Xiphinematobacter* were significantly decreased (*p* ≤ 0.05) in the on-year samples relative to those in the off-year samples (**Table 2**). Regarding the fungal communities (**Figure 3**; **Table 2**), compared to those in the off-year samples, the relative abundances of Ascomycota, *Mortierella*, *Hypocrea, Cryptococcus, Clitopilus*, and *Ceratocystis* in the on-year samples were significantly increased (*p* ≤ 0.05), whereas that of *Basidiomycota* was significantly decreased (*p* ≤ 0.05).

**Figure 4:**
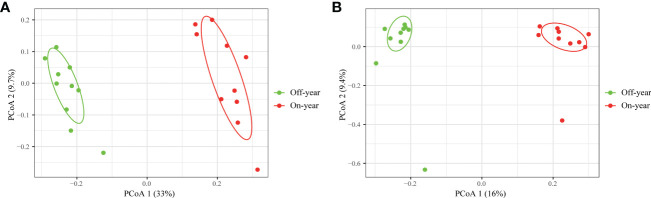
Principal coordinate analysis of soil bacterial **(A)** and fungal **(B)** communities in the on- and off-year Moso bamboo plantations.

PcoA was used to show the similarities and differences between the microbial taxa (**Figure 4**). Bacteria and fungi accounted for 42.7% and 25.4% of the variation on the first and second axis, respectively. Two different clusters were formed for the on- and off-year soil samples concerning the bacterial and fungal community compositions, indicating that on- and off-year management practices in the studied bamboo forest caused marked bacterial and fungal community shifts. ANOSIM also confirmed significant differences in the bacterial (R = 0.955, *p* = 0.001) and fungal communities (R = 0.707, *p* = 0.001) between the on- and off-year stands.

### 3.5 Correlations between soil environmental factors and bacterial and fungal community

**Figure 5:**
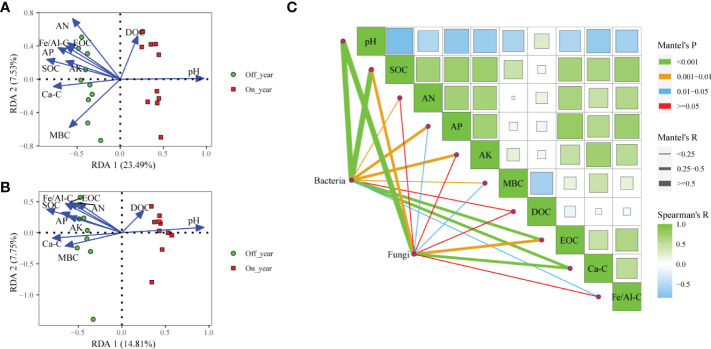
Redundancy analysis of soil bacterial **(A)** and fungal **(B)** communities and measured soil properties in the on- and off-year Moso bamboo forests. Spearman’s correlation analysis and Mantel tests for microbial communities **(C)**. SOC, soil organic C; AN, alkali-hydrolyzable N; AP, available P; AK, available K; MBC, microbial biomass C; DOC, dissolved organic C; EOC: easily oxidized organic C; Ca-C, Ca-bound soil organic C; Fe/Al-C, Fe/Al-bound soil organic C.

We conducted RDA and Mantel test to evaluate relationships between soil factors and microbial community structures (**Figure 5**). RDA showed that the first and second axes accounted for 31.02% of variance in bacterial communities (**Figure 5A**). Soil chemical parameters including pH, SOC, AN, AP, AK, MBC, DOC, EOC, Ca-SOC, and Fe/Al-SOC content significantly correlated with bacterial communities (r^2 =^ 0.910, 0.754, 0.797, 0.636, 0.430, 0.663, 0.304, 0.541, 0.588, and 0.531, respectively). The results of the Mantel test indicated that bacterial community structures significantly correlated with pH (r = 0.819, *p* ≤ 0.001), SOC (r = 0.533, *p* ≤ 0.001), AN (r = 0.232, *p* ≤ 0.01), AP (r = 0.337, *p* = 0.001), AK (r = 0.275, *p* = 0.003), MBC (r = 0.241, *p* = 0.007), EOC (r = 0.376, *p* ≤ 0.001), Ca-SOC (r = 0.411, *p* ≤ 0.001), and Fe/Al-SOC (r = 0.220, *p* = 0.011) (**Figure 5C**). Within the in fungal communities, the first two RDA axes accounted for 22.56% of variance (**Figure 5B**). Soil pH (r^2^ = 0.865, *p* = 0.001), SOC (r^2^ = 0.831, *p* = 0.001), AN (r^2^ = 0.453, *p* = 0.007), AP (r^2^ = 0.547, *p* = 0.002), AK (r^2^ = 0.416, *p* = 0.012), MBC (r^2^ = 0.458, *p* = 0.005), EOC (r^2^ = 0.551, *p* = 0.004), Ca-SOC (r^2^ = 0.622, *p* = 0.001), and Fe/Al-SOC (r^2^ = 0.594, *p* = 0.002) significantly affected fungal community structure. Furthermore, results of Mantel test suggested that pH (r = 0.562, *p* ≤ 0.001), SOC (r = 0.310, *p* = 0.002), AP (r = 0.186, *p* = 0.049), MBC (r = 0.241, *p* = 0.016), EOC (r = 0.339, *p* = 0.005), and Ca-SOC (r = 0.376, *p* = 0.001) had important effects on variations in fungal communities (**Figure 5C**).

**Figure 6:**
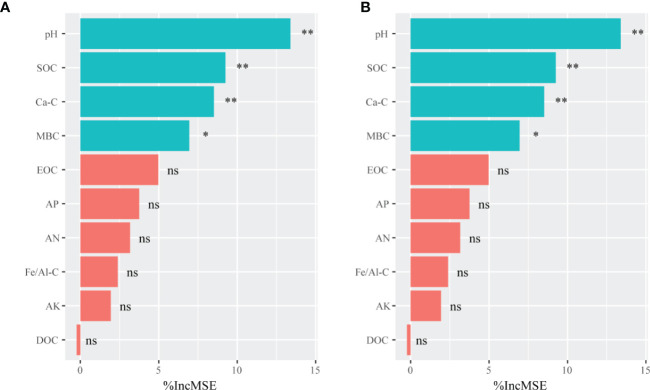
The randomForest analysis showing the relative contribution of soil properties in determining soil bacterial **(A)** and fungal **(B)** communities in the on- and off-year Moso bamboo forests. The bacterial and fungal community data represent alpha indices (Shannon and Chao1) and relative abundance of keystone taxa. **p ≤ 0.01; *p ≤ 0.05; ns, *p* > 0.05. %IncMSE: percentage of increased mean square error. SOC, soil organic C; Ca-C, Ca-bound soil organic C; MBC, microbial biomass C; EOC, easily oxidized organic C; AP, available P; AN, alkali-hydrolyzable N; Fe/Al-C, Fe/Al-bound soil organic C; AK, available K; DOC, dissolved organic C.

**Figure 7:**
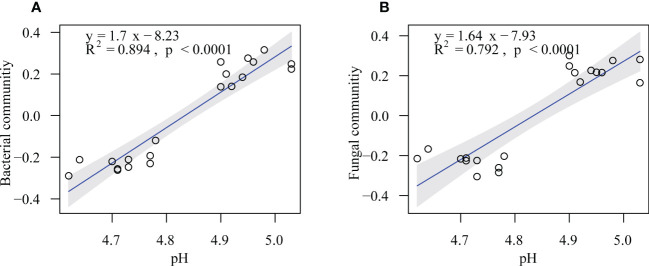
Soil pH in relation to soil bacterial **(A)** and fungal **(B)** communities in the on- and off-year Moso bamboo forests. Shaded areas show the 95% confidence interval of the fitted line.

The randomForest analysis was also conducted to identify the main soil factors responsible for bacterial and fungal communities. (**Figure 6**) Soil pH was the most important predictors of bacterial and fungal communities (percentages of increased mean square error (%IncMSE): 13.39% and 14.12%, respectively), followed by SOC (%IncMSE: 9.25% and 8.99%, respectively), Ca-C (%IncMSE: 8.51% and 8.20%, respectively), and MBC (%IncMSE: 6.95% and 6.40%, respectively). The linear model showed that the soil pH was positively correlated with bacteria (R^2^ = 0.894, *p* ≤ 0.0001) and fungi communities (R^2^ = 0.792, *p* ≤ 0.0001; **Figure 7**).

## 4 Discussion

### 4.1 Effect of on- and off-year management practices on SOC fractions

Large differences in the physiological and ecological traits of Moso bamboo forests between on- and off-year stands have been observed in several studies (Li et al., 1998; Volker and David, 2001; Gratani et al., 2008). Results showed that SOC, AN, and AP were significantly lower in the on-year Moso bamboo forest soil than in the off-year Moso bamboo forest soil, whereas the opposite trend was observed for AK (**Table 1**). This was probably because growing bamboo shoots in the on-year Moso bamboo stands mainly took up SOC, AN, and AP, whereas in the off-year Moso bamboo stands, AK was mainly used for replacing bamboo leaves and beeding bamboo shoots. Some studies have shown that SOC, N, and P play important roles in bamboo shoot growth and material growth in Moso bamboo, whereas K has significant effects on leaf growth (Wu et al., 2006; Song et al., 2016a).

C pools such as EOC, DOC, and MBC are sensitive to land-use type changes (Zhu et al., 2015; Gai et al., 2021). EOC was found to be the component with the highest content in SOC fractions in all bamboo soils as it has a slower turnover rate than that of other unstable C forms (Xu et al., 2010). Compared to those in the soil of off-year Moso bamboo stands, the EOC and MBC in the soil of on-year Moso bamboo stands decreased by 24.5% and 56.3%, respectively. Decreased soil EOC may be related to plant growth because it is highly available to plants (Xiao et al., 2021). McGill et al. (1986) showed that MBC can be used to represent SOC turnover and the nutrient cycle to a certain extent. Thus, MBC may be a sensitive indicator of SOC changes caused by the differences in on- and off-year management practices in Moso bamboo forests. The MBC is directly involved in soil biochemical transformation processes and is an important indicator of the role of soil microorganisms (Naorem et al., 2021). MBC/SOC has been often used as an indicator of how organic matter status changes land utilization (Ferreira et al., 2016). The MBC/SOC was 1.66% for off-year moso bamboo stands and 0.92% for on-year moso bamboo stands, with significant differences between them (p ≤ 0.05) (Table 1). This indicates that on- and off-year management practices change the structure of SOC fractions and promote the transformation of soil carbon, as well as indicating that the carbon sequestration capacity of soil microorganisms in off-year moso bamboo stands was significantly enhanced compared with that in on-year moso bamboo stands. However, in the present study, there was no significant difference between the DOC contents of on- and off-year stands, which may be related to the fact that DOC is the most active component of SOC and can be recovered in a short time after depletion (Singh et al., 2017).

SOC stability has an important relationship with its intermolecular chemical bonding (Kumada, 1987). Our results showed that the Fe/Al-SOC content in the studied soils was higher than the Ca-SOC content. This is because Fe/Al oxides are abundant in acidic soils od southern China and mainly form Fe/Al-bonded organic mineral complexes (Xu and Yuan, 1993b). Compared with those in the off-year stands, the Fe/Al-SOC and Ca-SOC contents in the on-year stands were significantly decreased. This indicated that the investigated Moso bamboo forest also consumed the passive organic C pool in the soil during bamboo shoot growth in the on-year stands, while the low growth of the Moso bamboo in the off-year stands facilitated the accumulation of passive organic C in the soil.

### 4.2 Effect of on- and off-year management practices on soil bacterial and fungal communities

A significant increase in both bacterial and fungal Chao1 and Shannon indices was observed in the on-year soils compared to those in the off-year soils (**Figure 2**), indicating that the on- and off-year management practices in Moso bamboo forests not only affected the diversity of soil bacteria and fungi, but also their species numbers. Meanwhile, PcoA showed that in the analysis of bacterial and fungal community compositions, two different clusters were formed for the on- and off-year soil samples (**Figure 4**). This indicated that the two management practices change the soil bacterial and fungal community compositions.

Our results showed that Acidobacteria and Proteobacteria were the dominant bacterial phyla in the soil of the study area (**Figure 3**, **5A, C**). Eichorst et al. (2018) showed that Acidobacteria can use various carbohydrates as C sources and also inorganic and organic N as N sources. Proteobacteria favor C-efficient soil and may promote the increase in organic C fractions and respiration (Fierer et al., 2007; Ren et al., 2018). These results suggested that the two dominant phyla play vital roles in the cycling of C and N in Moso bamboo plantations. However, the relative abundances of these two bacterial phyla did not differ significantly between the on- and off-year soils. The relative abundance of Actinobacteria was lower in the on-year soils than in the off-year soils (**Figure 3**). Some studies have shown that Actinobacteria play an influential role in nutrient cycling and organic matter decomposition (Narendrula-Kotha and Nkongolo, 2017; Pei et al., 2018). This indicates that these species promote nutrient cycling and depletion in off-year soils. We also found that the relative abundances of the genera *Acidothermus* and *Candidatus_Solibacter* (both within the phylum Acidobacteria) were also lower in the on-year soils than in the off-year soils (**Table 2** and **Figure 3**). This may be related to the harsh environmental conditions of the off-year soils. Most Actinobacteria species have a strong metabolic capacity and ability to rapidly colonize selective substrates (Narendrula-Kotha and Nkongolo, 2017), and some species have been shown to be resistant to acidic and other extreme environments (Barns et al., 2007).

Furthermore, results showed that Basidiomycota and Ascomycota were the dominant fungal phyla in the studied soils (**Figure 3**). Species belonging to these phyla are known to metabolize organic matter in forest litter and rhizosphere sediments, and their abundance is influenced by soil organic matter dynamics as a result of plant residue decomposition (Hannula et al., 2012; Bastida et al., 2013). The relative abundance of Basidiomycota in the soil was higher in the off-year stands than in the on-year stands, probably because of their strong ability to survive in soil environments with extreme pH or nutrient imbalances (Tedersoo et al., 2017), suggesting that soil quality actually improved during on-years. However, the relative abundances of *Cryptococcus* and *Clitopilus* within the phylum Basidiomycota were higher in the on-year soils than in the off-year soils. This is because the on-year period is when Moso bamboo plants require a lot of organic matter and nutrients to grow, whereas the genera *Cryptococcus* and *Clitopilus* have the ability to decay wood, break down plant and animal manure, and develop a symbiotic association with plant roots where they aid in the plant’s absorption of water, mineral salts, and metabolites, while also obtaining C and vital organic matter from the plant (Carris et al., 2012; Dejene et al., 2017). The relative abundance of Ascomycota was significantly higher in the off-year soils than in the on-year soils. Species belonging to this phylum are important decomposers in soils, as they can degrade litter containing refractory lignocellulose (Vandenkoornhuyse et al., 2002; Bastian et al., 2009; Angelini et al., 2012). Some Ascomycota species can decompose plant residues and use their easily degradable fraction to replenish soil C sources for their own growth and to promote the accumulation of C in the soil (Osono, 2007), as well as form a mutually reinforcing relationship with plants. The shifts in Basidiomycota and Ascomycota could explain the differences in SOC pools between the on- and off-year soils.

### 4.3 The mechanism of on- and off-year management practices affect soil SOC by changing soil microbial communities

Soil microbial communities can be characterized by their physicochemical properties (Rousk et al., 2010; Li and Liu, 2019; Zhang et al., 2022). The different plant growth status and related management measures in the on-year and off-year caused the change in soil physicochemical properties, consequently, resulted in the shift of soil microbial communities. In the present study, RDA, the Mantel test, and random forest analysis showed that the bacterial and fungal communities were mainly driven by soil pH and other available nutrients. Rousk et al. (2010) found that soil pH is correlated with bacterial and fungal communities. In this study, pH significantly shaped the microbial communities, meanwhile, the SOC fractions, SOC and Ca-SOC were negatively correlated with the soil pH (**Figure 5C**), indicating the pH might largely influence the SOC through the change of microbial communities and their activities. Previous studies have documented the effect of SOC on soil microbial communities (You et al., 2014; Paulina et al., 2020), and this effect was partly associated with the effect of SOC on other physicochemical properties (Gao et al., 2019). In this study, we found that the growth of Moso bamboo forest consumed a lot of soil nutrients in the on-year, which caused soil nutrients depletion and pH decrease at the beginning of the off-year. Actinobacterias and Basidiomycota could adapt to the harsh environmental conditions in the off-year and have the ability to decompose apoplastic matter, multiply (Narendrula-Kotha and Nkongolo, 2017; Tedersoo et al., 2017; Pei et al., 2018), and could make a large amount of apoplastic matter into organic matter and replenishing it into the soil, so that the soil SOC content of the bamboo forest can be greatly increased at the beginning of the on-year (Li and Liu, 2019; Song et al., 2021; Liu et al., 2022).

## 5 Conclusions

This study showed that off-year Moso bamboo forest management significantly increased the content of SOC and its fractions (EOC, MBC, Ca-SOC, and Fe/Al-SOC), indicating that this practice is beneficial for soil C sequestration. Furthermore, off-year management significantly decreased soil pH and AK, but increased AN and AP. The bacterial and fungal communities were significantly affected by on- and off-year management and best predicted by soil pH. The on- and off-year management measures enabled the nutrients and microbial communities in the Moso bamboo forest soil to be fully restored, which is of great significance for the sustainable management of Moso bamboo forests. Moreover, our findings revealed the characteristics of SOC fractions and microbial community composition in Moso bamboo soils under on- and off-year management practices. These results provide fundamental theoretical basis for the sustainable management and improvement of C sequestration in forest ecosystems.

## Data availability statement

The original contributions presented in the study are publicly available. This data can be found here: NCBI, PRJNA873135.

## Author contributions

Conceptualization, XZ and ZZ. Methodology, XZ. Investigation, XZ, ZH, QL, XG, FB, and CY. Writing—original draft preparation, ZH. Writing—review and editing, XZ and ZZ. Funding acquisition, XZ and ZZ. All authors contributed to the article and approved the submitted version.
