# Corrigendum: Effects of on- and off-year management practices on the soil organic C fractions and microbial community in a Moso bamboo (*Phyllostachys edulis*) forest in subtropical China

**DOI:** 10.3389/fpls.2022.1121575

**Published:** 2023-01-12

**Authors:** Zhiyuan Huang, Qiaoling Li, Xu Gai, Xiaoping Zhang, Zheke Zhong, Fangyuan Bian, Chuanbao Yang

**Affiliations:** ^1^ China National Bamboo Research Center, Key Laboratory of Bamboo Forest Ecology and Resource Utilization of National Forestry and Grassland Administration, Hangzhou, Zhejiang, China; ^2^ National Long-term Observation and Research Station for Forest Ecosystem in Hangzhou-Jiaxing-Huzhou Plain, Hangzhou, Zhejiang, China; ^3^ Key Laboratory of High Efficient Processing of Bamboo of Zhejiang Province, Hangzhou, Zhejiang, China; ^4^ Research Institute of Subtropical Forestry, Chinese Academy of Forestry, Hangzhou, Zhejiang, China; ^5^ Engineering Research Center of Biochar of Zhejiang Province, Hangzhou, Zhejiang, China

**Keywords:** Bamboo, on- and off-year, C sequestration, bacteria, fungi

**Text Correction**

In the published article, there was an error in **2 Materials and methods**, “*2.5 Bacterial and fungal communities analysis”*. This sentence previously stated: “The 341F-805R (5′-CCTACGGG?>NGGCWFLASH (Magoc and Salzberg, 2011) was used to construct paired-end 16s and ITS1 sequences, which were subsequently quality-trimmed and length-filtered using Fqtrim.”

The corrected sentence appears below:

“The 341F-805R (5′-CCTACGGGNGGCWGCAG-3′/5′-GACTACHVGGGTATCTAATCC-3′) and ITS1FI2-ITS2 (5′-GAACCWGCGGARGGATCA-3′/5′-GCTGCGTTCTTCATCGATGC-3′) primer sets were used to amplify the bacterial 16S V3-V4 region and fungal ITS2 genes. Amplicon synthesis, library construction, and Illumina NovaSeq sequencing (2 × 250 bp) were performed by LC-Bio Technology Co., Ltd. (Hangzhou, China). FLASH (Magoc and Salzberg, 2011) was used to construct paired-end 16s and ITS1 sequences, which were subsequently quality-trimmed and length-filtered using Fqtrim.”

In the published article, there was an error in **4 Discussion**, *“4.1 Effect of on- and off-year management practices on SOC fractions*”. This sentence previously stated: “The MBC/SOC was 1.65% for off-year moso bamboo stands and 0.93% for on-year moso bamboo stands, with significant differences between them (p ≤ 0.05).”

The corrected sentence appears below:

“The MBC/SOC was 1.66% for off-year moso bamboo stands and 0.92% for on-year moso bamboo stands, with significant differences between them (p ≤ 0.05) (Table 1).”

The authors apologize for these errors and state that this does not change the scientific conclusions of the article in any way. The original article has been updated.

